# A nomogram for predicting viral encephalitis based on cerebrospinal fluid biomarkers

**DOI:** 10.3389/fneur.2026.1769471

**Published:** 2026-03-27

**Authors:** Xinhui Yu, Shichao Gao, Yaomeng Huang, Xiaotong Shen, Shuai Zhao, Jing Chen, Jingna Sun

**Affiliations:** 1 Hebei Medical University, Shijiazhuang, Hebei, China; 2 Department of Clinical Laboratory, The First Hospital of Hebei Medical University, Shijiazhuang, Hebei, China

**Keywords:** biomarker, cerebrospinal fluid, inflammatory cytokines, nomogram, predictive model, viral encephalitis

## Abstract

**Background:**

VE is a central nervous system infection of viral origin, and remains an important disease burden in recent years. Early identification of VE patients and timely interventions are crucial for optimizing clinical outcomes.

**Objective:**

This study aims to construct a predictive model for early detection of VE patients.

**Methods:**

The study retrospectively analyzed clinical data of 160 VE and 131 non-VE patients from China between January 2022 and March 2025. Data were split into training (70%, 203 cases) and validation (30%, 88 cases) cohorts. Predictor variables were identified via logistic regression analyses, and predictive models were established and validated. Model discrimination was assessed using ROC curves, calibration via H-L test and calibration curves, and clinical applicability via DCA. A nomogram was developed for result visualization.

**Results:**

Six covariates (ALB, *β*_2_-MG, IFN-*γ*, CSF WBC, CSF LYM, CSF P) were identified as independent VE risk factors (OR >1), and IFN-*α* as a protective factor (OR <1). In the training cohort, a visualized nomogram was built based on the stepwise multivariate logistic regression. AUC of the nomogram in the training and validation cohorts was 0.86 (95% CI, 0.80–0.91) and 0.85 (95% CI, 0.77–0.93). The calibration curves for the probability of VE infection showed optimal agreement between prediction by nomogram and actual observation. DCA indicated that nomogram conferred high clinical net benefit.

**Conclusion:**

This study’s predictive model reliably identifies VE patients, offering a scientific basis for clinical decision-making and improving patient outcomes.

## Introduction

Viral encephalitis (VE) is an acute inflammatory disease of the central nervous system (CNS), involving the brain parenchyma and meninges (1). It can be caused by various pathogens, including Herpes Simplex Virus (HSV), Varicella-Zoster Virus (VZV), enteroviruses, and Japanese Encephalitis Virus (JEV) (2). These viruses invade the body through different routes, cross the blood–brain barrier, replicate within the CNS, and trigger inflammatory responses, leading to brain injury and functional disorders. The clinical manifestations of VE are often non-specific, with common initial symptoms such as fever, headache, nausea, and vomiting. As the disease progresses, some patients may develop neurological and psychiatric symptoms such as epileptic seizures, personality changes, and behavioral abnormalities. Severe cases can result in permanent neurological deficits, including dementia, epilepsy, and hemiplegia (3). Despite active treatment, most patients are left with varying degrees of neurological deficits, which places a heavy burden on patients and their families (4).

VE is the most common CNS infection worldwide, with significant variations in epidemiological characteristics depending on factors such as age and geographical location (5, 6). In the United States, there are approximately 6,000 VE-related hospitalizations annually, with total medical costs reaching as high as $540 million (7). In Asia, Japanese encephalitis is the primary pathogen causing VE, with an estimated 67,900 to 100,000 cases occurring each year, 75% of which are in children under the age of 14 (8). Although the incidence of JE has declined due to widespread vaccination, it remains prevalent during epidemic seasons. A study conducted in sentinel hospitals across Shandong, Hubei, Guangxi, and Hebei provinces (all located in eastern China) from 2006 to 2007 revealed that 9.2% of acute meningitis and encephalitis patients were diagnosed with Japanese encephalitis (9).

Currently, the diagnosis of VE primarily relies on viral nucleic acid testing of cerebrospinal fluid, such as polymerase chain reaction (PCR) and next-generation sequencing (NGS) (10). While these technologies offer high sensitivity and specificity, they have limitations in terms of being time-consuming and costly, which can result in patients missing the optimal treatment window. Early identification of VE patients and timely intervention are crucial for improving the clinical outcomes of VE patients.

Recent research has increasingly focused on identifying diagnostic markers and developing predictive models for VE. Diagnostic markers for acute encephalitis syndrome and COVID-19-associated multisystem inflammatory syndrome in pediatric populations have been identified in Southern India (11). Additionally, the diagnostic accuracy of inflammatory markers in suspected central nervous system infections among adults has been systematically evaluated (12). Further studies have demonstrated that cerebrospinal fluid (CSF) biomarkers related to brain injury, neuroinflammation, and synaptic autoimmunity may serve as predictors of long-term neurocognitive outcomes in herpes simplex encephalitis (13). A recent predictive model incorporating PD-1 and ICOS has also been proposed to facilitate early differentiation between autoimmune encephalitis and VE (14). However, these studies often focus on single markers or specific patient groups, lacking comprehensive multi-factor analysis and the construction of broadly applicable models. Despite these advances, existing studies often rely on single biomarkers or are limited to specific patient cohorts, lacking a comprehensive multi-factor analysis. Current models integrating multiple CSF biomarkers for early VE diagnosis remain limited.

Given this, the present study aims to analyze the clinical data of VE patients to construct and validate a multi-factorial logistic regression prediction model. By incorporating a variety of potential markers, this model is designed to provide clinicians with a comprehensive and efficient tool for the early identification of VE patients, thereby enhancing treatment decisions and improving patient prognoses.

## Materials and methods

### Study population

**Figure 1:**
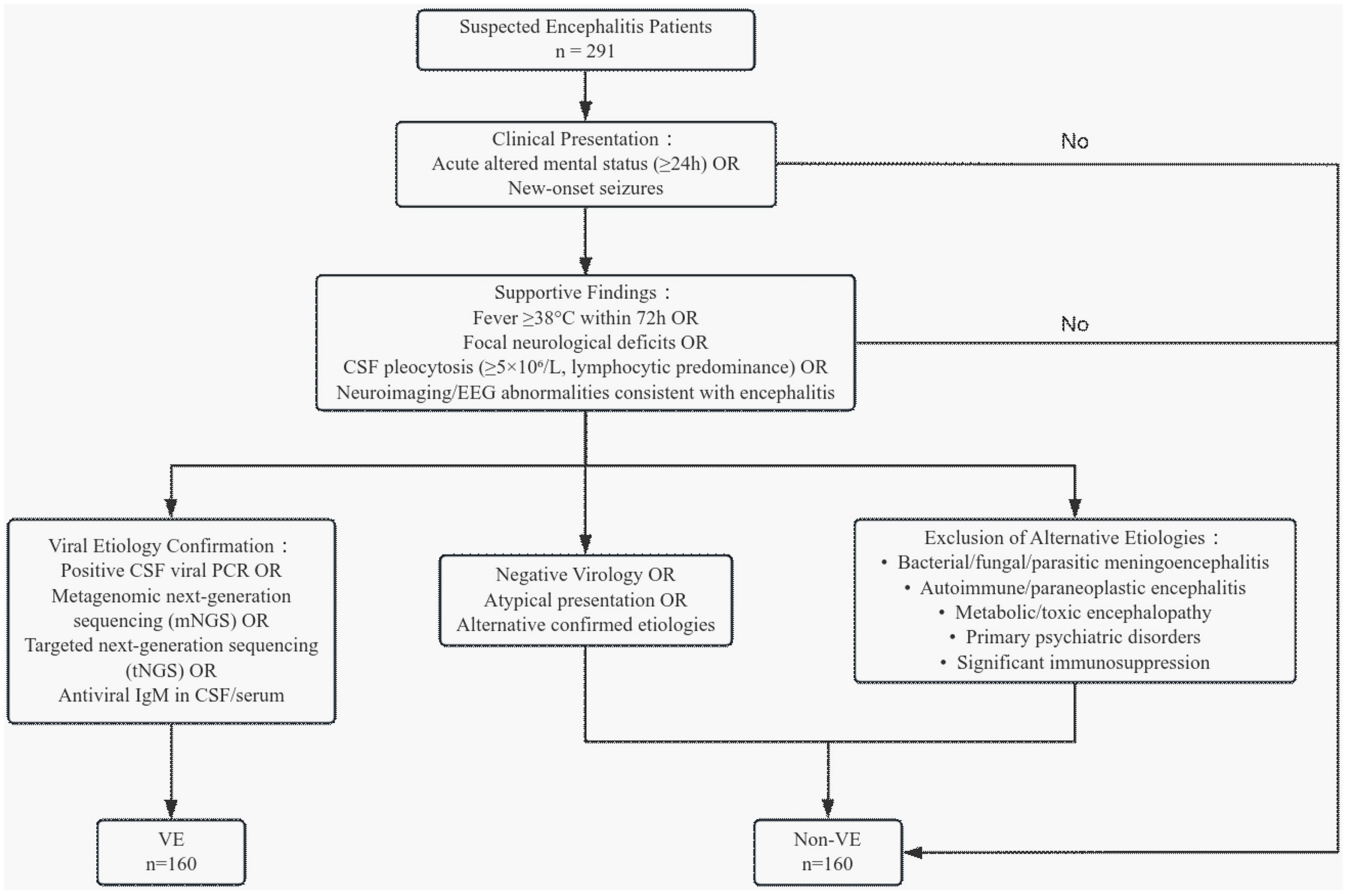
Diagnostic flowchart for patient enrollment and group allocation.

This retrospective study enrolled 160 patients with VE and 131 with non-VE at the First Hospital of Hebei Medical University (March 2022–March 2025). VE diagnostic criteria comprised four mandatory components: (1) acute altered mental status ≥24 h or new-onset seizures; (2) fever ≥38 °C within 72 h, focal neurological deficits, CSF pleocytosis (≥5 × 10^6^/L, lymphocytic predominance), or neuroimaging/EEG abnormalities consistent with encephalitis; (3) positive CSF viral PCR, metagenomic next-generation sequencing (mNGS), targeted next-generation sequencing (tNGS), or antiviral IgM in CSF/serum; (4) systematic exclusion of alternative etiologies (15, 16). Diagnostic criteria for non-viral encephalitis (control group): patients with any of the following—negative virology (PCR, mNGS, tNGS, IgM); atypical presentation deemed indeterminate by board-certified neurologists; laboratory-confirmed alternative infections (bacterial/fungal/parasitic meningoencephalitis); autoimmune/paraneoplastic encephalitis; metabolic/toxic encephalopathy or primary psychiatric disorders; or significant immunosuppression (advanced AIDS, solid organ transplantation, active chemotherapy). The diagnostic flowchart is presented in Figure 1.

### Methodologies

By accessing our clinical electronic medical record system and hospital information system, we retrieved and collected the clinical data and indicators of the patients at the time of admission. These included gender, age, infection-related symptoms (such as fever, headache, nausea, vomiting, and impaired consciousness, etc.), meningeal irritation signs, hypertension, albumin (ALB), *β*_2_-MG, procalcitonin (PCT), blood leukocyte counts (WBCs), neutrophil ratios (NEU), lymphocyte ratio (LYM), neutrophil-to-lymphocyte ratio (NLR), Interferon-alpha (INF-*α*), Interferon-gamma (INF-*γ*), interleukin 6 (IL-6), C-reactive protein (CRP), cerebrospinal fluid leukocytes (CSF WBC), cerebrospinal fluid lymphocytes (CSF LYM), cerebrospinal fluid protein (CSF P), cerebrospinal fluid chloride (CSF CL), and cerebrospinal fluid glucose (CSF G). Continuous variables were initially examined in their original scale; however, non-significant linear associations and non-normal distributions led to median-based categorization for model stability (Table 1).

**Table 1:** Value assignments of indicators in the viral encephalitis diagnostic prediction model.

Indicators	Assignment
ALB	≥ 47.5 g/L = 0, < 47.5 g/L = 1
*β*_2_-MG	≤ 2 mg/L = 0, > 2 mg/L = 1
PCT	≤ 0.25 ng/mL = 0, > 0.25 ng/mL = 1
WBC	≤ 6.5 × 10^9^/L = 0, > 6.5 × 10^9^/L = 1
NEU	≥ 57.5% = 0, < 57.5% = 1
LYM	≤ 35% = 0, > 35% = 1
NLR	≥ 2 = 0, < 2 = 1
IFN-*α*	≤ 4.25 pg./mL = 0, > 4.25 pg./mL = 1
IFN-*γ*	≤ 3.71 pg./mL = 0, > 3.71 pg./mL = 1
IL-6	≤ 3.5 pg./mL = 0, > 3.5 pg./mL = 1
CRP	≤ 5 mg/L = 0, > 5 mg/L = 1
CSF LYM	≤ 50% = 0, > 50% = 1
CSF WBC	≤ 4 × 10^6^/ L = 0, > 4 × 10^6^/ L = 1
CSF P	≤ 300 mg/L = 0, > 300 mg/L = 1
CSF CL	≤ 126 mmol/L = 0, > 126 mmol/L = 1
CSF G	≥ 3.5 mmol/L = 0, < 3.5 mmol/L = 1

ALB, Albumin; *β*_2_-MG, Beta-2 Microglobulin; PCT, Procalcitonin; WBC, White Blood Cell count; NEU, Neutrophil; LYM, Lymphocyte; NLR, Neutrophil to Lymphocyte Ratio; INF-*α*. Interferon-alpha; INF-*γ*, Interferon-gamma; IL-6, Interleukin-6; CRP, C-Reactive Protein; CSF LYM, Cerebrospinal Fluid Lymphocyte; CSF WBC, Cerebrospinal Fluid White Blood Cell; CSF P, Cerebrospinal Fluid Protein; CSF CL, Cerebrospinal Fluid Chloride; CSF G, Cerebrospinal Fluid Glucose.

### Statistical analysis

In this study, data analysis and visualization were performed using Z-stats and R software. The dataset was randomly divided into training and validation cohorts at a 7:3 ratio. Descriptive statistics were first conducted for all variables in the training cohort. Continuous variables were assessed for normality using the Shapiro–Wilk test. Normally distributed data were expressed as mean ± standard deviation and analyzed using t-tests, whereas non-normally distributed data were presented as median and interquartile range [M (*P*25, *P*75)] and compared via the Mann–Whitney U test. Categorical variables were summarized as frequencies and percentages (*n*, %), with intergroup differences evaluated using the chi-square test or Fisher’s exact test.

Variables demonstrating significant differences were further subjected to multivariate logistic regression analysis via bidirectional stepwise regression to identify independent predictors of VE diagnosis. A nomogram was constructed for visualization. The model’s predictive performance was evaluated using the receiver operating characteristic (ROC) curve and area under the curve (AUC), with an AUC > 0.85 indicating excellent discriminative ability. Goodness-of-fit was confirmed by a non-significant Hosmer-Lemeshow test (H-L test) (*p* > 0.05) and further validated by calibration curves. Decision curve analysis (DCA) was applied to assess clinical net benefit.

All statistical tests were two-sided, with *p* < 0.05 considered statistically significant, except for the H-L test.

## Result

### Comparison of baseline characteristics between training and validation cohorts in VE patients

This study comprised 291 patients in total. Of these, 70% were randomly designated to the training cohort (203 patients) and 30% to the validation cohort (88 patients). The training cohort included 88 males and 115 females, with a mean age of 38.90 ± 21.53 years. The validation cohort consisted of 42 males and 46 females, with a mean age of 35.68 ± 19.26 years. Comparative analysis of baseline characteristics between the two groups revealed no statistically significant differences (*p* > 0.05) (Supplementary Table S1).

### Differential analysis of baseline data for VE patients in training cohort

The differential analysis of baseline data in the training cohort was performed among 203 patients, consisting of 89 (43.84%) in the non-VE group and 114 (56.16%) in the VE group. Statistically significant differences were observed between the two groups in terms of ALB, *β*_2_-MG, WBC, INF-*α*, INF-*γ*, CSF LYM, CSF WBC, and CSF P (*p* < 0.05), while no statistically significant differences were found in age, gender, infection-related symptoms, meningeal irritation signs, hypertension, PCT, NEU, LYM, NLR, IL-6, CRP, CSF CL, and CSF G (*p* > 0.05) (Table 2).

**Table 2:** Comparison of baseline characteristics of 203 viral encephalitis patients in the training cohort.

Indicators	VE (*n* = 114)	Non-VE (*n* = 89)	*t*/*χ*^2^	*p*
Age, years, mean ± SD	38.71 ± 22.22	39.13 ± 20.73	0.14	0.89
Gender, *n* (%)			1.71	0.19
Male	54 (47.37)	34 (38.20)		
Female	60 (52.63)	55 (61.80)		
Infection-related symptoms, *n* (%)			0.52	0.47
No	32 (28.07)	21 (23.60)		
Yes	82 (71.93)	68 (76.40)		
Meningeal irritation, *n* (%)			0.45	0.50
No	73 (64.04)	61 (68.54)		
Yes	41 (35.96)	28 (31.46)		
Hypertension, *n* (%)			0.48	0.49
No	82 (71.93)	60 (67.42)		
Yes	32 (28.07)	29 (32.58)		
ALB, *n* (%)			22.51	**< 0.01**
≥ 47.5 g/L	10 (8.77)	32 (35.96)		
< 47.5 g/L	104 (91.23)	57 (64.04)		
*β*_2_-MG, *n* (%)			12.26	**< 0.01**
≤ 2 mg/L	41 (35.96)	54 (60.67)		
> 2 mg/L	73 (64.04)	35 (39.33)		
PCT, *n* (%)			0.64	0.42
≤ 0.25 ng/ mL	76 (66.67)	64 (71.91)		
> 0.25 ng/ mL	38 (33.33)	25 (28.09)		
WBC, *n* (%)			4.19	**0.04**
≤ 6.5 × 10^9^/ L	54 (47.37)	55 (61.80)		
> 6.5 × 10^9^/ L	60 (52.63)	34 (38.20)		
NEU, *n* (%)			0.13	0.72
≥ 57.5%	77 (67.54)	58 (65.17)		
< 57.5%	37 (32.46)	31 (34.83)		
LYM, *n* (%)			0.47	0.49
≤ 35%	88 (77.19)	65 (73.03)		
> 35%	26 (22.81)	24 (26.97)		
NLR, *n* (%)			0.35	0.56
≥ 2	75 (65.79)	55 (61.80)		
< 2	39 (34.21)	34 (38.20)		
INF-*α*, *n* (%)			9.97	**< 0.01**
≤ 4.25 pg./ mL	98 (85.96)	60 (67.42)		
> 4.25 pg./ mL	16 (14.04)	29 (32.58)		
INF-*γ*, *n* (%)			11.45	**< 0.01**
≤ 3.71 pg./ mL	51 (44.74)	61 (68.54)		
> 3.71 pg./ mL	63 (55.26)	28 (31.46)		
IL-6, *n* (%)			0.59	0.44
≤ 3.5 pg./ mL	7 (6.14)	8 (8.99)		
> 3.5 pg./ mL	107 (93.86)	81 (91.01)		
CRP, *n* (%)			0.52	0.47
≤ 5 mg/ L	38 (33.33)	34 (38.20)		
> 5 mg/ L	76 (66.67)	55 (61.80)		
CSF LYM, *n* (%)			8.05	**< 0.01**
≤ 50%	7 (6.14)	17 (19.10)		
> 50%	107 (93.86)	72 (80.90)		
CSF WBC, *n* (%)			38.42	**< 0.01**
≤ 4 × 10^6^/ L	59 (51.75)	82 (92.13)		
> 4 × 10^6^/ L	55 (48.25)	7 (7.87)		
CSF P, *n* (%)			12.95	**< 0.01**
≤ 300 mg/ L	39 (34.21)	53 (59.55)		
> 300 mg/ L	75 (65.79)	36 (40.45)		
CSF CL, *n* (%)			0.49	0.48
≤ 126 mmol/ L	62 (54.39)	44 (49.44)		
> 126 mmol/ L	52 (45.61)	45 (50.56)		
CSF G, *n* (%)			1.30	0.25
≥ 3.5 mmol/ L	63 (55.26)	42 (47.19)		
< 3.5 mmpl/ L	51 (44.74)	47 (52.81)		

*t*, t-test; *χ*^2^, Chi-square test. ALB, Albumin; *β*_2_-MG, Beta-2 Microglobulin; PCT, Procalcitonin; WBC, White Blood Cell count; NEU, Neutrophil; LYM, Lymphocyte; NLR, Neutrophil to Lymphocyte Ratio; INF-*α*. Interferon-alpha; INF-*γ*, Interferon-gamma; IL-6, Interleukin-6; CRP, C-Reactive Protein; CSF LYM, Cerebrospinal Fluid Lymphocyte; CSF WBC, Cerebrospinal Fluid White Blood Cell; CSF P, Cerebrospinal Fluid Protein; CSF CL, Cerebrospinal Fluid Chloride; CSF G, Cerebrospinal Fluid Glucose. Significant differences (*p* < 0.05) are shown in bold.

### Univariate and multivariate logistic regression analyses for VE

**Figure 2:**
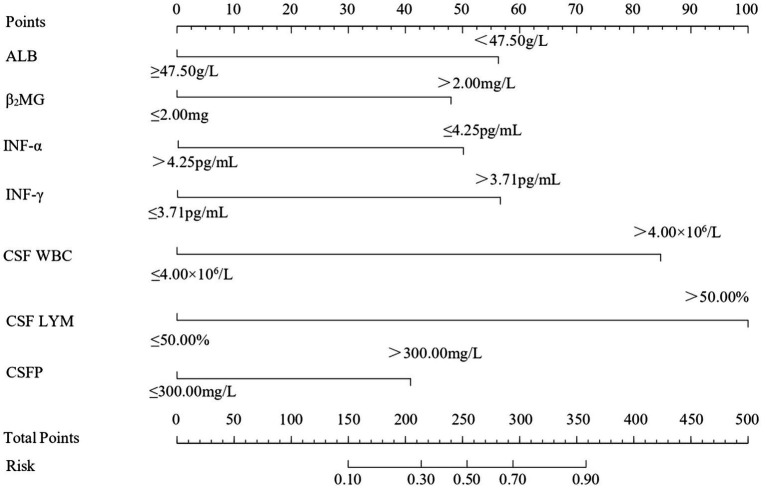
Nomogram for predicting the risk of VE based on multiple logistic regression analysis of independent risk factors. The nomogram includes the following variables: Albumin (ALB), Beta-2 Microglobulin (*β*_2_-MG), Interferon-alpha (INF-*α*), Interferon-gamma (INF-*γ*), Cerebrospinal Fluid White Blood Cell Count (CSF WBC), Cerebrospinal Fluid Lymphocyte Percentage (CSF LYM), and Cerebrospinal Fluid Protein (CSFP). Each variable is assigned a specific point value based on its level, which is then summed to obtain the total points. The total points are then used to estimate the probability of VE on the risk scale at the bottom of the nomogram.

Univariate logistic regression analysis revealed that ALB, *β*_2_-MG, WBC, INF-*α*, INF-*γ*, CSF LYM, CSF WBC, and CSF P were statistically significant predictors (*p* < 0.05). To further identify VE predictors, we conducted a multifactorial logistic regression analysis. The final model demonstrated excellent discriminative ability (Nagelkerke *R*^2^ = 0.452) and highly significant overall fit (likelihood ratio *χ*^2^ = 83.56, df = 6, *p* < 0.01). The results showed that ALB had an OR of 3.26 (95% CI: 1.34–7.96, *p* < 0.01), *β*_2_-MG had an OR of 2.74 (95% CI: 1.34–5.57, *p* < 0.01), INF-*α* had an OR of 0.34 (95% CI: 0.14–0.82, *p* = 0.02), INF-*γ* had an OR of 3.25 (95% CI: 1.53–6.93, *p* < 0.01), CSF LYM had an OR of 8.05 (95% CI: 2.36–27.37, *p* < 0.01), CSF WBC had an OR of 6.24 (95% CI: 2.17–17.96, *p* < 0.01), and CSF P had an OR of 2.37 (95% CI: 1.11–5.08, *p* = 0.03). These findings indicate that ALB, *β*_2_-MG, INF-*γ*, CSF LYM, CSF WBC, and CSF P are independent risk factors for VE (OR > 1), while INF-*α* is a protective factor (OR < 1). Based on these seven indicators, we constructed a multifactorial logistic regression model: ln [*p*/(1-*p*)] = −4.16 + 1.18 × ALB + 1.01 × *β*_2_-MG − 1.08 × INF-*α* + 1.18 × INF-*γ* + 2.09 × CSF LYM + 1.83 × CSF WBC + 0.86 × CSF P. A nomogram was plotted for visualization (Table 3; Figure 2).

**Table 3:** Univariate and multivariate logistic regression analyses for VE and non-VE patients.

Indicators	Univariate analysis			Multivariate analysis		
	*β*	OR (95%CI)	*p*	*β*	OR (95%CI)	*p*
ALB, *n* (%)						
< 47.5 g/ L	1.76	5.84 (2.68 ~ 12.74)	**< 0.01**	1.18	3.26 (1.34 ~ 7.96)	**< 0.01**
*β*_2_-MG, *n* (%)						
> 2 mg/ L	1.01	2.75 (1.55 ~ 4.87)	**< 0.01**	1.01	2.74 (1.34 ~ 5.57)	**< 0.01**
WBC, *n* (%)						
> 6.5 × 10^9^/ L	0.59	1.80 (1.02 ~ 3.16)	**0.04**			
INF-*α*, *n* (%)						
> 4.25 pg./ mL	−1.09	0.34 (0.17 ~ 0.67)	**< 0.01**	−1.08	0.34 (0.14 ~ 0.82)	**0.02**
INF-*γ*, *n* (%)						
> 3.71 pg./ mL	0.99	2.69 (1.51 ~ 4.81)	**< 0.01**	1.18	3.25 (1.53 ~ 6.93)	**< 0.01**
CSF LYM, *n* (%)						
> 50%	1.28	3.61 (1.42 ~ 9.14)	**< 0.01**	2.09	8.05 (2.36 ~ 27.37)	**< 0.01**
CSF WBC, *n* (%)						
> 4 × 10^6^/ L	2.39	10.92 (4.65 ~ 25.67)	**< 0.01**	1.83	6.24 (2.17 ~ 17.96)	**< 0.01**
CSF P, *n* (%)						
> 300 mg/ L	1.04	2.83 (1.60 ~ 5.02)	**< 0.01**	0.86	2.37 (1.11 ~ 5.08)	**0.03**

OR, Odds Ratio; CI, Confidence Interval. Variables with *p*-values < 0.2 in the univariate logistic regression analysis were subsequently included in a multivariate stepwise bidirectional logistic regression model. ALB, Albumin; *β*_2_-MG, Beta-2 Microglobulin; WBC, White Blood Cell count; INF-*α*, Interferon-alpha; INF-*γ*, Interferon-gamma; CSF LYM, Cerebrospinal Fluid Lymphocyte; CSF WBC, Cerebrospinal Fluid White Blood Cell; CSF P, Cerebrospinal Fluid Protein. Significant differences (*p* < 0.05) are shown in bold.

### Multicollinearity assessment

Multicollinearity among the seven predictor variables was assessed using generalized variance inflation factor (GVIF). Conventionally, GVIF< 5 or tolerance> 0.2 indicates no severe multicollinearity. In our final model, all GVIF values ranged from 1.10 to 1.65, with tolerance ranging from 0.61 to 0.91 (Table 4).

**Table 4:** Multicollinearity assessment of predictor variables in the final model.

Indicators	Tolerance	GVIF
CSF WBC	0.61	1.65
CSF CL	0.77	1.29
CSF P	0.78	1.28
ALB	0.79	1.27
IFN-*γ*	0.86	1.16
IFN-*α*	0.87	1.16
*β*_2_-MG	0.91	1.10

GVIF, generalized variance inflation factor; All GVIF values well below 5 indicate no severe multicollinearity.

### Evaluating the discrimination of predictive models

**Figure 3:**
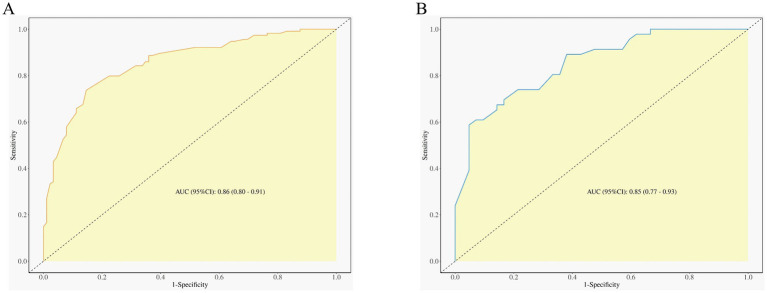
Discrimination for the nomogram in the training and validation cohorts. **(A)** Training cohort and **(B)** validation cohort. ROC, Receiver operating characteristic. AUC, area under the curve.

In clinical practice, ROC curves are commonly used to evaluate a model’s discriminatory ability. In this study, the ROC curve for the training cohort demonstrated an AUC of 0.86 (95%CI: 0.80–0.91). The optimal cutoff value was 0.56, achieving an accuracy of 79%, sensitivity of 85%, and specificity of 74% (Table 5; Figure 3A), indicating strong accuracy in distinguishing between viral and non-VE patients. Similarly, the validation cohort’s ROC curve showed an AUC of 0.85 (95%CI: 0.77–0.93) with the same cutoff value. Here, accuracy was 76%, sensitivity 79%, and specificity 74% (Table 5; Figure 3B).

**Table 5:** Performance measures of the viral encephalitis diagnostic prediction model.

Index	Training cohort (*n* = 203)	Validation cohort (*n* = 88)
AUC (95%CI)	0.86 (0.80–0.91)	0.85 (0.77–0.93)
Accuracy (95%CI)	0.79 (0.73–0.84)	0.76 (0.66–0.85)
Sensitivity (95%CI)	0.85 (0.78–0.93)	0.79 (0.66–0.91)
Specificity (95%CI)	0.74 (0.66–0.82)	0.74 (0.61–0.87)

CI, Confidence Interval; AUC, area under the curve.

### Evaluating the calibration of predictive models

**Figure 4:**
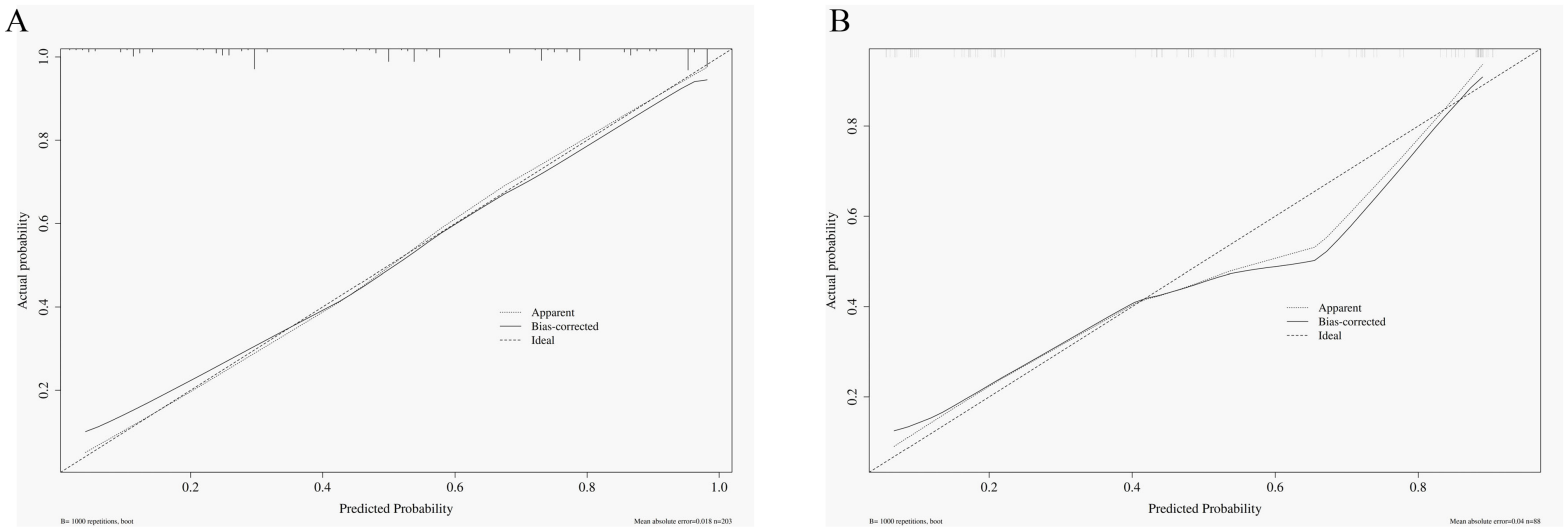
Calibration curves for the nomogram in the training and validation cohorts. **(A)** Training cohort; **(B)** Validation cohort. Apparent: shows the unadjusted calibration curve of the model. Bias-corrected: represents the calibration curve after applying bias correction techniques, aiming for better accuracy. Ideal: Represents perfect calibration, where the predicted probability exactly matches the observed probability. The closer the ‘Apparent’ and ‘Bias-corrected’ lines are to the ‘Ideal’ line, the better the calibration.

To assess model stability and calibration, we performed bootstrap internal validation with 1,000 resamples and plotted calibration curves for both cohorts. The training cohort showed excellent calibration [mean absolute error (MAE) = 0.018; AUC = 0.86], with predicted probabilities closely aligning with observed outcomes (H-L test, *p* = 0.58). The validation cohort demonstrated robust performance (MAE = 0.040; AUC = 0.85) with good calibration (H-L test, *p* = 0.89). Calibration plots (Figures 4A,B) showed tight clustering around the ideal diagonal line, with calibration slope slope close to 1 and intercept close to 0, indicating minimal overfitting and reliable predictive accuracy across both datasets.

### Clinical utility assessment of predictive models

**Figure 5:**
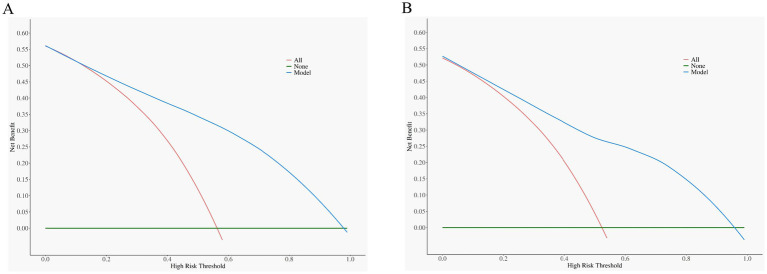
DCA for the evaluation of the clinical applicability of the nomogram. The blue line represents the net benefit. Both in the **(A)** training and **(B)** validation cohort, the nomogram yields clinical net benefits when the threshold probability is between 15 and 65%. These results indicate good potential for clinical utility. DCA, decision curve analysis.

DCA was performed to evaluate the model’s net benefit across clinically relevant risk thresholds. In the training cohort (Figure 5A), the model demonstrated positive net benefit compared to both the “treat-all” and “treat-none” strategies when the high-risk threshold ranged from approximately 15 to 70%. Within this range, the model achieved a net benefit of 0.10–0.35, indicating its potential to guide clinical decision-making by appropriately identifying patients requiring further diagnostic workup. The validation cohort (Figure 5B) showed consistent results, with the model maintaining positive net benefit across a similar threshold range (15–65%). Beyond these ranges, the net benefit approached zero as the threshold approached extreme values, consistent with expected DCA behavior.

### Etiological results

Among 160 patients with viral encephalitis, 140 underwent pathogen-targeted next-generation sequencing (100 + panel). Seventy-nine cases tested positive, with a positivity rate of 56.43% (79/140), while 61 cases were negative.

In the 79 positive cases, the distribution of viral pathogens was as follows: herpes simplex virus type 1 (*n* = 30, 37.97%), Epstein–Barr virus (*n* = 13, 16.46%), varicella-zoster virus (*n* = 13, 16.46%), influenza A virus (*n* = 7, 8.86%), enterovirus (*n* = 6, 7.59%), human herpesvirus 7 (*n* = 3, 3.80%), herpes simplex virus type 2 (*n* = 2, 2.53%), cytomegalovirus (*n* = 2, 2.53%), human herpesvirus 6 (*n* = 2, 2.53%), and adenovirus (*n* = 1, 1.27%) (Table 6).

**Table 6:** Distribution and constituent ratio of viral pathogens in 79 positive cases.

Viral pathogen	Number (*n* = 79)	Proportion (%)
Herpes simplex virus type 1	30	37.97
Epstein–Barr virus	13	16.46
Varicella-zoster virus	13	16.46
Influenza A virus	7	8.86
Enterovirus	6	7.59
Human herpesvirus 7	3	3.8
Herpes simplex virus type 2	2	2.53
Cytomegalovirus	2	2.53
Human herpesvirus 6	2	2.53
Adenovirus	1	1.27

## Discussion

This study constructed a predictive model for VE through a comprehensive multi-indicator analysis, encompassing both serum markers (such as ALB, *β*_2_-MG, IFN-*α*, and IFN-*γ*) and cerebrospinal fluid cytological markers (such as CSF WBC, CSF LYM, and CSF P). This represents the first integration of multiple serum and CSF markers into a single predictive model. Previous studies have explored diagnostic markers for VE or related neurological conditions, but several limitations exist in the existing literature. For example, one investigation focused on diagnostic biomarkers for acute encephalitis syndrome but was limited to pediatric populations within specific geographic regions (11). Another study evaluated inflammatory markers in adult central nervous system infections but did not develop a multi-indicator predictive model (12). In contrast, the present study not only established a comprehensive nomogram incorporating CSF biomarkers but also rigorously validated its performance using both training and independent validation cohorts, thereby enhancing the model’s stability and clinical applicability. Notably, existing predictive models for neurological infections frequently lack external validation, compromising their generalizability. A representative case is the recently developed model for differentiating autoimmune encephalitis from VE in early-stage presentations (14), which demonstrated promising discriminative performance but was limited by the absence of independent cohort validation, thereby constraining its clinical applicability. Our study found no significant differences in clinical symptoms between VE and non-VE patients (*p* < 0.05), underscoring the critical importance of analyzing laboratory indicators.

VE is a central nervous system infection triggered by viruses, involving complex immune and inflammatory responses. ALB, primarily synthesized by the liver, reflects liver function and blood–brain barrier integrity. In VE, inflammation may suppress hepatic ALB synthesis, reducing serum ALB levels (17). Meanwhile, increased blood–brain barrier permeability causes serum ALB to leak into the cerebrospinal fluid, further lowering serum ALB levels (18). Accumulating evidence have demonstrated that the cerebrospinal fluid/serum ALB ratio (QAlb) is a key indicator for assessing blood–brain barrier permeability and is closely linked to blood–brain barrier disruption in herpes simplex virus type 1 encephalitis (19).

*β*_2_-MGs, light chains of MHC class I molecules, are widely present on nucleated cell surfaces. Viral infection-induced inflammation activates the immune system, causing immune cell proliferation and increased *β*_2_-MG release (20). During viral replication, the virus damages host cells, releasing intracellular *β*_2_-MG into the bloodstream and elevating serum *β*_2_-MG levels (21).

IFN-*α* and IFN-*γ* are crucial pro-inflammatory immune factors with antiviral effects. IFN-*α* quickly limits viral spread in the early infection phase (22), while IFN-*γ* enhances natural killer cell and CD8 + T cell cytotoxicity in the late phase (23). In this study, IFN-*α*’s protective role reflects its importance in early antiviral responses, whereas elevated IFN-*γ* may indicate disease progression and tissue damage. Pro-inflammatory cytokines also boost the antiviral immune response by increasing blood–brain barrier permeability and facilitating immune cell entry into the CNS (24). These indicators reflect the body’s immune response to viral infection. Once the immune system is activated, LYM and WBC in the cerebrospinal fluid infiltrate the CNS, aiding virus clearance but also causing inflammation and brain tissue damage. This leads to plasma albumin leakage into the cerebrospinal fluid and elevated CSF P levels, which are positively correlated with VE and show potential as early diagnostic biomarkers (25). Consequently, CSF LYM, CSF WBC, and CSF P levels rise significantly. CSF levels of WBC, LYM, and CSF P directly mirror the CNS’s inflammatory and immune response. Tyler KL highlighted the link between cerebrospinal fluid cells and VE in 2018 (26). These indicators are vital for VE diagnosis and closely related to disease severity and prognosis. Given the severe and potentially irreversible consequences of VE, monitoring these indicators and predicting VE patients through modeling is crucial for clinical supportive diagnosis (27).

The model demonstrated robust discrimination in both the training and validation cohorts, with AUC values of 0.86 and 0.85, respectively. In comparison, other studies have reported lower model performance. Notably, a previous model utilizing CSF biomarkers to predict long-term neurocognitive outcomes in herpes simplex encephalitis achieved an AUC of 0.78 (13), which was inferior to the discriminative performance of our nomogram. Additionally, we assessed the calibration of our model using the H-L test and calibration curves, revealing high consistency between predicted probabilities and actual observations (*p* = 0.58 for the training cohort and *p* = 0.89 for the validation cohort). Some studies have not conducted a detailed evaluation of calibration, which may compromise the accuracy of their models in real-world applications. We also used DCA to assess how much our model actually helps clinicians at different risk thresholds. DCA confirmed the model’s utility as an auxiliary diagnostic aid across clinically relevant probability thresholds (15–65%), guiding clinicians to prioritize confirmatory testing—including lumbar puncture and pathogen-targeted assays—for patients with elevated predicted likelihood of viral encephalitis. In contrast, other studies have predominantly focused on the statistical performance of their models, neglecting their practical value in clinical decision-making. Compared with other risk factors, such as positive CSF viral tests (28), the indicators included in our model are more accessible and convenient, thereby enhancing its real-world applicability. Our model not only exhibits excellent performance and interpretability but also shows strong discrimination, calibration, and clinical utility in diagnosing VE. To the best of our knowledge, this is the first diagnostic prediction model for VE.

While significant progress has been made in developing joint diagnostic indicators for other diseases, providing more precise clinical diagnostic tools and methods (29, 30), the research on joint diagnostic indicators for VE remains in its infancy and has yet to achieve a major breakthrough. Currently, VE diagnosis predominantly relies on single indicators or methods, such as cerebrospinal fluid examination, imaging studies, or molecular biological testing (31). These approaches have limitations in specificity or sensitivity. For instance, traditional virus isolation methods, though considered the “gold standard” (32), are complex, time-consuming, and have low sensitivity, particularly during the early infection stage, making them difficult to apply widely in clinical settings. Molecular biological techniques like PCR and mNGS have shown high sensitivity and specificity in pathogen detection (33), but they are costly, require strict laboratory conditions, and may yield false-negative results at low viral loads (34). Moreover, while some studies have attempted to construct encephalitis prognostic prediction models based on clinical symptoms through multifactorial logistic regression analyses, the metrics included in these models are relatively limited and homogeneous (35). The prediction model developed in this study incorporates CSF biomarkers and blood parameters routinely measured in central nervous system infection workups, enabling prompt risk stratification once laboratory results become available. The model functions as a screening instrument to triage patients for definitive virological confirmation. By flagging individuals who warrant urgent lumbar puncture and pathogen-targeted assays, it streamlines diagnostic workflow and optimizes resource utilization. This approach integrates host immune response markers with pathophysiological indicators to support clinical decision-making.

Although the model demonstrated favorable predictive performance, several limitations warrant consideration. This single-center retrospective study was limited by modest sample size and internal split validation—rather than true external validation—potentially constraining generalizability. Additionally, median-based dichotomization was necessitated by non-significant linear associations and non-normal distributions of continuous CSF variables. Future multicenter prospective studies with larger cohorts should validate this model in independent external settings, employing LASSO or elastic net regression and exploring machine learning approaches to enhance predictive accuracy.

## Conclusion

This study developed and validated a multivariate logistic regression model incorporating ALB, *β*_2_-MG, INF-*α*, INF-*γ*, and CSF biomarkers (WBC, LYM, and Protein) for early identification of VE patients. The model demonstrated robust discriminative performance and clinical applicability, offering clinicians a practical tool to early identify VE and guide therapeutic decision-making. Future studies should further validate its generalizability across diverse populations and investigate additional diagnostic markers to refine predictive accuracy and expand clinical utility.

## Data Availability

The datasets presented in this study can be found in online repositories. The names of the repository/repositories and accession number(s) can be found in the article/Supplementary material.

## Glossary

VE: Viral Encephalitis
CSF: Cerebrospinal Fluid
ALB: Albumin
*β*_2_-MG: *β*_2_-microglobulin
PCT: Procalcitonin
WBC: White Blood Cell
NEU: Neutrophil
LYM: Lymphocyte
NLR: Neutrophil to Lymphocyte Ratio
IL-6: Interleukin-6
CRP: C-Reactive Protein
IFN-*α*: Interferon-alpha
IFN-*γ*: Interferon-gamma
CSF LYM: Cerebrospinal Fluid Lymphocyte
CSF WBC: Cerebrospinal Fluid White Blood Cell
CSF P: Cerebrospinal Fluid Protein
CSF CL: Cerebrospinal Fluid Chloride
CSF G: Cerebrospinal Fluid Glucose
OR: Odds Ratio
CI: Confidence Interval
AUC: Area under the curve
ROC: Receiver operating characteristic
DCA: Decision curve analysis
