# Supplementary material for: A nomogram for predicting viral encephalitis based on cerebrospinal fluid biomarkers
Source: Front Neurol. 2026 Mar 27;17:1769471. doi: 10.3389/fneur.2026.1769471 (PMC13065507; doi:10.3389/fneur.2026.1769471)
Supplement: Supplementary file 1 [file Table_1.docx]

Table S1. Comparison of baseline information between training and validation cohorts

| **Indicators** | **Validation cohort (n = 88)** | **Training cohort (n = 203)** | **t / χ²** | ***P*** |
| --- | --- | --- | --- | --- |
| Age, years, mean ± SD | 35.68 ± 19.26 | 38.90 ± 21.53 | -1.26 | 0.21 |
| Gender, n (%) |  |  | 0.48 | 0.49 |
| Male | 42 (47.73) | 88 (43.35) |  |  |
| Female | 46 (52.27) | 115 (56.65) |  |  |
| Infection-related symptoms, n (%) |  |  | 2.82 | 0.09 |
| No | 15 (17.05) | 53 (26.11) |  |  |
| Yes | 73 (82.95) | 150 (73.89) |  |  |
| Meningeal irritation, n (%) |  |  | 0.87 | 0.35 |
| No | 63 (71.59) | 134 (66.01) |  |  |
| Yes | 25 (28.41) | 69 (33.99) |  |  |
| Hypertension, n (%) |  |  | 0.76 | 0.38 |
| No | 57 (64.77) | 142 (69.95) |  |  |
| Yes | 31 (35.23) | 61 (30.05) |  |  |
| ALB, n (%) |  |  | 0.07 | 0.79 |
| ≥ 47.5 g/ L | 17 (19.32) | 42 (20.69) |  |  |
| < 47.5 g/ L | 71 (80.68) | 161 (79.31) |  |  |
| β_2_-MG, n (%) |  |  | 0.11 | 0.75 |
| ≤ 2 mg/ L | 43 (48.86) | 95 (46.80) |  |  |
| > 2 mg/ L | 45 (51.14) | 108 (53.20) |  |  |
| PCT, n (%) |  |  | 0.41 | 0.52 |
| ≤ 0.25 ng/ mL | 64 (72.73) | 140 (68.97) |  |  |
| > 0.25 ng/ mL | 24 (27.27) | 63 (31.03) |  |  |
| WBC, n (%) |  |  | 1.24 | 0.27 |
| ≤ 6.5 × 10^9^/ L | 41 (46.59) | 109 (53.69) |  |  |
| > 6.5 × 10^9^/ L | 47 (53.41) | 94 (46.31) |  |  |
| NEU, n (%) |  |  | 1.10 | 0.29 |
| ≥ 57.5 % | 64 (72.73) | 135 (66.50) |  |  |
| < 57.5 % | 24 (27.27) | 68 (33.50) |  |  |
| LYM, n (%) |  |  | 0.12 | 0.73 |
| ≤ 35 % | 68 (77.27) | 153 (75.37) |  |  |
| > 35 % | 20 (22.73) | 50 (24.63) |  |  |
| NLR, n (%) |  |  | 0.01 | 0.90 |
| ≥ 2 | 57 (64.77) | 130 (64.04) |  |  |
| < 2 | 31 (35.23) | 73 (35.96) |  |  |
| INF-α, n (%) |  |  | 3.04 | 0.08 |
| ≤ 4.25 pg/ mL | 60 (68.18) | 158 (77.83) |  |  |
| > 4.25 pg/ mL | 28 (31.82) | 45 (22.17) |  |  |
| INF-γ, n (%) |  |  | 0.19 | 0.66 |
| ≤ 3.71 pg/ mL | 51 (57.95) | 112 (55.17) |  |  |
| > 3.71 pg/ mL | 37 (42.05) | 91 (44.83) |  |  |
| IL-6, n (%) |  |  | 0.65 | 0.42 |
| ≤ 3.5 pg/ mL | 9 (10.23) | 15 (7.39) |  |  |
| > 3.5 pg/ mL | 79 (89.77) | 188 (92.61) |  |  |
| CRP, n (%) |  |  | 0.27 | 0.61 |
| ≤ 5 mg/ L | 34 (38.64) | 72 (35.47) |  |  |
| > 5 mg/ L | 54 (61.36) | 131 (64.53) |  |  |
| CSF LYM, n (%) |  |  | 0.19 | 0.67 |
| ≤ 50 % | 12 (13.64) | 24 (11.82) |  |  |
| > 50 % | 76 (86.36) | 179 (88.18) |  |  |
| CSF WBC, n (%) |  |  | 0.17 | 0.68 |
| ≤ 4 × 10^6^/ L | 59 (67.05) | 141 (69.46) |  |  |
| > 4 × 10^6^/ L | 29 (32.95) | 62 (30.54) |  |  |
| CSF P, n (%) |  |  | 0.77 | 0.38 |
| ≤ 300 mg/ L | 35 (39.77) | 92 (45.32) |  |  |
| > 300 mg/ L | 53 (60.23) | 111 (54.68) |  |  |
| CSF CL, n (%) |  |  | 0.52 | 0.47 |
| ≤ 126 mmol/ L | 50 (56.82) | 106 (52.22) |  |  |
| > 126 mmol/ L | 38 (43.18) | 97 (47.78) |  |  |
| CSF G, n (%) |  |  | 0.65 | 0.42 |
| ≥ 3.5 mmol/ L | 41 (46.59) | 105 (51.72) |  |  |
| < 3.5 mmpl/ L | 47 (53.41) | 98 (48.28) |  |  |

t, t-test; χ², Chi-square test. ALB, Albumin; β_2_-MG, Beta-2 Microglobulin; PCT, Procalcitonin; WBC, White Blood Cell count; NEU, Neutrophil; LYM, Lymphocyte; NLR, Neutrophil to Lymphocyte Ratio; INF-α. Interferon-alpha; INF-γ, Interferon-gamma; IL-6, Interleukin-6; CRP, C-Reactive Protein; CSF LYM, Cerebrospinal Fluid Lymphocyte; CSF WBC, Cerebrospinal Fluid White Blood Cell; CSF P, Cerebrospinal Fluid Protein; CSF CL, Cerebrospinal Fluid Chloride; CSF G, Cerebrospinal Fluid Glucose.
